# Oesophageal atresia is correctable and survivable in infants less than 1 kg

**DOI:** 10.1007/s00383-015-3851-4

**Published:** 2016-04-18

**Authors:** Edward J. Hannon, Jennifer Billington, Edward M. Kiely, Agostino Pierro, Lewis Spitz, Kate Cross, Joseph I. Curry, Paolo De Coppi

**Affiliations:** Department of Paediatric and Neonatal Surgery, Great Ormond Street Hospital, London, UK; Stem Cells and Regenerative Medicine Section, Developmental Biology and Cancer Programme, UCL Institute of Child Health, 30 Guilford Street, London, WC1N 1EH UK

**Keywords:** Oesophageal atresia, Trachea-oesophageal fistula, Extremely low birth weight

## Abstract

**Introduction:**

Management of oesophageal atresia (OA) and trachea-oesophageal fistula (TOF) in babies of low birth weight is challenging especially when associated with other anomalies. Birth weight of <1500 g has previously formed part of a classification system designed to predict outcome, alongside the cardiac status of the patient. Improvements in neonatal care have led to increasing numbers of premature low birth weight infants surviving. The aim of this study was to look at the experience of our institution in the extremely low birth weight (ELBW) patients.

**Methods:**

A retrospective review of our institutions OA database was performed from 1993 to June 2015. Patients of birth weight less than 1000 g were included. A review of our OA/TOF clinical database and notes review established the following; gestation, birth weight, associated anomalies, operative procedures, morbidity and mortality.

**Results:**

Of 349 patients with OA across the 22-year period, 9 ELBW patients were identified (<1000 g). Six males and three females. Gestational age ranged from 23 to 34 weeks and median birth weight was 815 g ranging from 630 to 950 g. Overall survival was 56 % (5/9). There were double the numbers of ELBW OA/TOF patients seen in the second half of the study period presumably the result of improving neonatal care. Seven patients had type C OA with TOF and underwent emergency TOF ligation, two had concomitant oesophageal repair. One of these patients died from NEC; the other survived. Of the five who had isolated TOF ligation three died—two from cardiac disease and one from prematurity. Both type A patients survived and after initial gastrostomy placement one had a primary delayed repair, the other a gastric transposition. All three babies under 800 g died—one from cardiac disease the others from conditions indicative of their prematurity—necrotising enterocolitis and intraventricular haemorrhage.

**Conclusions:**

50 % survival is achievable in OA/TOF under 1 kg and the Spitz classification is still applicable in this group as a whole. However, none of the current classification systems are applicable in infants <800 g who in our study all had poor outcomes. We suggest these should be considered as separate group when predicting outcomes.

## Introduction

Oesophageal atresia (OA) with or without tracheoesophageal fistula (TOF) occurs in 1 in 3000–4500 live births and survival of these patients has improved over the last 30 years [1]. The principle of surgery for these cases remains the early repair of any TOF and primary or delayed repair of the oesophagus. The only significant change to this surgical management over the last 10 years has been the advent of the minimally invasive approach. However, improvements in pre and postoperative neonatal care and anaesthetic care, especially of low birth weight and premature babies have led to improved outcomes in all neonates including those with OA/TOF [2–4].

In attempts to predict survival, Spitz classified OA/TOF patients into three groups according to birth weight (>1500 g) and the presence of major cardiac defects [5]. This classification has been broadly adopted when reporting OA/TOF outcomes and has changed practice in some centres which advocate early TOF ligation alone in babies less than 1500 g with delayed OA repair in attempts to minimise early morbidity and mortality [6]. More recent attempts to reclassify outcome in light of improving neonatal care have suggested different weight limits than Spitz [7] or scoring systems which take into account other associated anomalies [8]. However, none of these studies have specifically focussed on the very low birth weight population.

With the improvement in neonatal care we have seen increasing numbers of very low (VLBW) and extremely low birth weight (ELBW) babies in our centre. We therefore wanted to review our experience of managing this ‘high-risk’ group of patients, focussing on operative strategies, co-morbidities, classification and survival.

## Methods

A retrospective review of our departmental OA/TOF patient database (1993–June 2015) was performed in order to identify all ELBW patients (defined as birth weight <1000 g). This database was created from hospital diagnostic coding data, cross checked with neonatal discharge summaries and surgeon log books to minimise error. This would therefore include any cases which died before surgical intervention. Each of the cases identified were then reviewed using the database, electronic records and clinical notes review. The following outcomes were identified: birth weight and gestation, presence of a cardiac defect, other associated abnormalities, operative approach, morbidity and mortality including cause of death.

A major cardiac defect was defined as per Spitz [5] as cyanotic disease or non-cyanotic disease requiring surgery or medical treatment for heart failure. A minor cardiac defect was defined as any other cardiac disease, including patient ductus arteriosus (PDA) and atrial septal defect (ASD). In addition, the presence of VACTERL, chromosomal abnormalities and other congenital anomalies were noted. Survival was defined as survival to discharge from hospital. Patients identified were subsequently classified using the Spitz classification for comparison of survival rates.

Statistical analysis was not performed on the results given the low number of cases.

## Results

In total 349 cases of OA/TOF were found across the 22-year study period, 9 (six males) were identified as being ELBW babies. In these nine patients gestational age ranged from 23 to 34 weeks and median birth weight was 815 g ranging from 630 to 950 g (Table 1). There were two cases from twin pregnancies. The incidence of cases across the time period is shown in Table 2. This demonstrates the increasing numbers of ELBW babies seen as a percentage of all OA/TOF babies in the second half of the study period with double the number of cases and double the proportion of all OA/TOF patients under 1000 g. Overall survival was 56 % (5/9) and was associated to the type of OA. There were no survivors in patients weighing <800 g.

**Table 1:** Summary of nine patients <1 kg

Patient number and year	Birth weight (g)	Gestation (weeks)	Type	Major cardiac defect	Associated anomalies	Primary procedure	Delayed definitive surgery	Survived (cause of death)
1 2013	630	31	C	Yes co-arctation of aorta		Thoracotomy, TOF ligation and gastrostomy	n/a	No—cardiac
2 2014	660	23	C	No (PDA)		Complete primary repair	n/a	No—NEC
3 1996	750	25	C	No	Polycystic kidney/renal failure	Thoracotomy, TOF ligation and gastrostomy	n/a	No—IVH
4 2000	800	34	C	No (PDA)	Duodenal atresia	Repair of gastric perforation, duodenoduodenostomy and TOF repair (another centre)	Ligation of recurrent fistula and oesophageal repair (3 months)	Yes
5 2009	815	34	C	Yes tetralogy of fallot	VACTERL, 13 ribs, bilateral hydronephrosis, ambiguous genitalia	Thoracotomy, TOF ligation and gastrostomy	n/a	No—Cardiac
6 2008	870	29	A	No		Gastrostomy insertion	Delayed primary repair—thoracoscopic converted to open (26 months old)	Yes
7 2008	890	29 + 6	C	Yes tetralogy of fallot	CHARGE syndrome, hemi vertebrae, renal dysplasia	Thoracotomy, TOF ligation and gastrostomy	Delayed OA repair (2 months old)	Yes
8 1995	905	31	A	No		Gastrostomy insertion	Delayed gastric transposition (10 months old)	Yes
9 2014	950	32 + 1	C	No	Duodenal atresia	Complete primary repair + duodenoduodenostomy	n/a	Yes

**Table 2:** Comparison of case load during the study period

Time period	Total OA/TOF patient	<1000 g patients	Percentage of patients <1000 g (%)
1993–2003	189	3	1.6
2004–2015	160	6	3.8

**Fig. 1:**
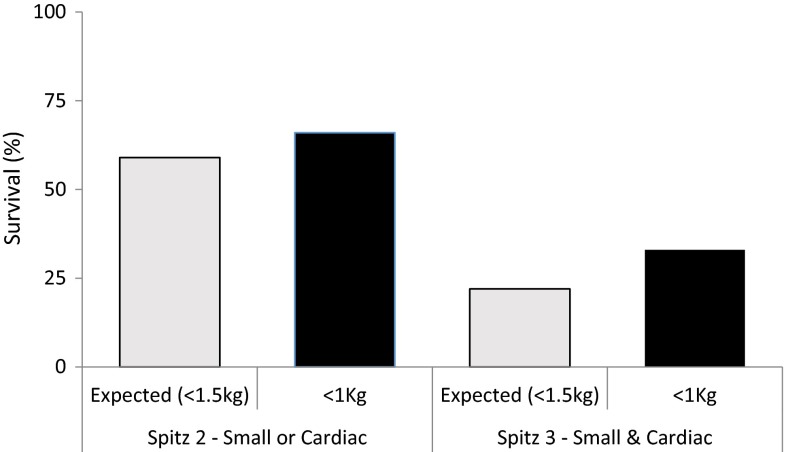
Comparison between expected survival by Spitz classification and observed survival in babies with OA <1 kg

When classified according to Spitz classification all patients were either in group 2 or 3 given their birth weights of under 1500 g. Those without a major cardiac defect were therefore in group 2 and with a major cardiac defect classified as group 3. Figure 1 compares our results in less than 1 kg babies to the original expected survival from Spitz classification (<1500 g babies). It suggests broadly similar or slightly improved survival in our cohort compared to Spitz’s expected survival, despite ours only including the ELBW patients.

The majority of cases (7/9; 78 %) were type C-OA with a distal fistula. Two (22 %) had primary complete repairs and 5 (56 %) underwent primary ligation of fistula (one in another centre) and gastrostomy insertion. Of the two who had complete primary repair case number 9 (950 g) also required duodenoduodenostomy for duodenal atresia and aortopexy for tracheomalacia and is well at 1-year follow up. The other died from NEC 1 week after repair. Of the five who initially had isolated TOF ligation, only two survived to definitive repair and both are still alive. Case 4 required redo fistula repair having had initial surgery in another centre. Both cases of type A-OA survived and were treated initially with gastrostomy insertion in the neonatal period—one in another institution. Delayed gap assessment allowed one infant to have a primary anastomosis at 20 months of age and the other a gastric transposition at 9 months of age and both are still alive.

The three smallest patients, all less than 800 g, did not survive. One died as a result of congenital cardiac disease, one from intra-ventricular haemorrhage (IVH), and one from necrotising enterocolitis (NEC). The latter (patient 2) underwent a primary repair on day 1 of life, but died from NEC 1 week post-operatively. At post mortem the oesophageal anastomosis and fistula repair were both intact.

## Discussion

Our results of OA/TOF in babies under 1000 g confirm the difficulty in managing small babies with OA, with a survival of 55 % compared to the 95 % expected survival in babies of >1500 g [9]. However, the results also demonstrate the quality of contemporary neonatal care as survival in the most high risk group—those with associated cardiac disease, is still over 30 %, even in infants under 1000 g. This increased survival of premature infants over the last two decades is supported by the increasing numbers of cases of ELBW infants seen across the study period—as increasingly premature, smaller babies are surviving to the point of surgical consultation.

Several authors have devised or revised classification systems to predict survival specifically in OA/TOF to give parents accurate details of prognosis and allow fair comparison of results between centres. Since Waterson [10] in 1963 through Spitz [9] and more recently Turner’s ‘contemporary’ approach [8] at classification, we see different ways to predict outcome. Our findings suggest Spitz’s original model for classification and survival is applicable in the <1000 g patients with broadly similar survival in the moderate risk (59 vs 66 %) and high-risk (22 vs 33 %) groups—again highlighting the significance of major cardiac defects in these patients.

Interestingly the most recent scoring system designed by Turner et al. [8], which combines a scoring system for each of the following; birth weight <1500 g, renal abnormality, cardiac abnormality and chromosomal abnormality would only have classified one of our patients as high risk due to the weighting towards renal disease and chromosomal anomaly in their scoring system.

The most significant finding from our study is the poor survival under 800 g. Whilst this may be expected when looked at from a purely neonatal perspective, such a poor survival would not be predicted using any of the classification/scoring systems in the literature [11]. According to Spitz [9] or Turner [8], two of the patients weighing <800 g in our series would have been in an intermediate risk group with expected survival of nearly 60 %. However, independent of their cardiac status, there was no survivors born <800 g. Whilst one of these was a cardiac death, the other two were both from conditions which represented the prematurity of the patients (necrotizing enterocolitis and intra ventricular haemorrhage). The expected survival for infants of similar gestation without OA/TOF has been estimated to be around 26 % at 23 weeks and up to 72 % at 25 weeks [12].We did not have any survivors below 25 weeks suggesting the significant effect of OA/TOF and surgical intervention on survival at these gestations This relatively new population of extremely premature babies (23–25 weeks) in our study obviously has an impact on survival and has not been previously looked at as a subgroup of patients. Further larger series are needed to confirm this finding, which could be relevant when counselling families with very premature babies born with OA.

Whilst accurate classification is useful for predicting outcomes, of more practical importance to the surgeon is the discussion around the surgical management and decision making for such patients in the acute post-natal setting. The group with OA and TOF may require emergency ligation of the TOF in order to achieve stability; however, the discussion around the oesophageal repair is more difficult. The risk of prolonging an anaesthetic, on a single lung in a potentially unstable small baby and performing a difficult anastomosis has to be balanced against the benefit of early oesophageal continuity and avoidance of further surgery.

Over the 22-year study period there have obviously been changes in neonatal, anaesthetic and surgical care and individual surgeons that have influenced the decision making processes. Our centre’s current management of ELBW or VLBW infants with OA is on a case by case basis. In the presence of a stable baby, without risk factors for prolonged anaesthetic, such as extreme prematurity or unstable heart disease a primary oesophageal anastomosis may be attempted. In our study complete repair and oesophageal anastomosis was attempted in two children—one with good outcome, the other (660 g) died from NEC 1 week post operatively—with an intact oesophagus. Seitz supports the use of primary anastomosis in VLBW and ELBW babies with OA/TOF in the absence of other significant anomalies but was based on a small series of four cases [13].

Any baby with OA/TOF in our unit which is not thought stable enough for the prolonged anaesthetic needed for complete primary repair or who deteriorates during surgery, will have a staged approach. If very unstable the initial fistula ligation can be done on the neonatal intensive care unit. This involves a standard right extra-pleural thoracotomy which can be converted to trans-pleural if more rapid access is required. If the patient is very unstable a simple ligation of the fistula is performed. This option, however, is associated to a very high early recurrence of the fistula and therefore is not the treatment choice at our Institution. Whenever possible, the TOF is divided and over sewn and the distal oesophagus is closed with non-absorbable sutures. In case an anastomosis is not achieved, a stamm type gastrostomy is then usually placed via an upper mini-laparotomy (usually midline). If necessary the gastrostomy can be delayed until the patient is more stable and can be maintained on total parenteral nutrition.

Careful replogle suction is required until oesophageal repair but can be difficult in the low birth weight infants. Timing of definitive oesophageal repair is again done on an individual patient basis and is usually related to comorbidities for example the possible need for cardiac surgery. We generally favour repairing the oesophagus as early as the patient’s condition allows to minimise the risk of re-fistulation and reduce the risk of aspiration. Routine staged repair in the low birth weight OA/TOF patients has been encouraged since the early result of primary repair in smaller infants was poor [14]. Alexander et al. in 1993 supported staged repair in babies less than 2000 g based on a study of 25 cases with higher complication rates in those who had primary repair [14]. A study by Petrosyan et al. from Los Angeles in 1993, also included 25 cases but in patients of lower birth weight of <1500 g. They concluded delayed repair of the oesophagus should be the routine at <1500 g due to higher incidence of post-operative complications and increased risk of stricturing with primary repair. The numbers of patients in our series obviously limits meaningful comparison to Petrosyan’s study but our experience in case 9 of successful primary anastomosis in a 950 g baby without complication and good outcomes at 1 year of age, suggest a blanket policy of delayed repair will result in some patients getting unnecessary staged surgery. We also have concern about the risk of re-fistulation in those who have initial isolated TOF ligation as they wait for definitive oesophageal repair. Therefore in a stable patient we will usually attempt repair. Our experience in very low birth weight babies also supports this individual approach to management.

It was interesting to find that both patients with type A atresia survived. This may be related to the lack of associated anomalies in both of these patients but may also be the fact that by nature of their pure OA they do not undergo the morbidity of an emergency thoracotomy on the first day of life. Both could have early gastrostomy insertion and delayed oesophageal repair or replacement.

Our experience of ELBW OA/TOF patients is limited by the small number of patients, but represent one of the larger series of OA/TOF under 1 kg. The progresses of neonatal care, the increasing survival of small babies with significant anomalies, including major heart defects, means this population will increase. The long duration of our study period is a result of the rarity of these cases and whilst it makes interpreting the data more difficult it demonstrates well the increasingly frequency of the ELBW infants in our practice over the time period.

## Conclusions

We have shown that survival of over 50 % is achievable in OA/TOF patients under 1 kg and this is probably testimony to the increasing standards of neonatal care. Spitz classification is still applicable across the <1 kg group as a whole but Spitz or any of the other classification systems published may not applicable to babies under 800 g, who in our small study had a poor outcome. Further data are needed to confirm these finding, however we suggest these babies are classified in a separate group and parents are counselled accordingly of the poor prognosis.
